# Thermodynamics of Ga_2_O_3_ Heteroepitaxy and Material Growth Via Metal Organic Chemical Vapor Deposition

**DOI:** 10.1021/acsaelm.4c00535

**Published:** 2024-06-21

**Authors:** Indraneel Sanyal, Arpit Nandi, David Cherns, Martin Kuball

**Affiliations:** Center for Device Thermography and Reliability, H. H. Wills Physics Laboratory, University of Bristol, Tyndall Avenue, Bristol BS81TL, United Kingdom

**Keywords:** Gallium oxide, Ultrawide bandgap, Ga_2_O_3_ epitaxy, Heteroepitaxy of Ga_2_O_3_, Thermodynamic model, MOCVD growth of Ga_2_O_3_

## Abstract

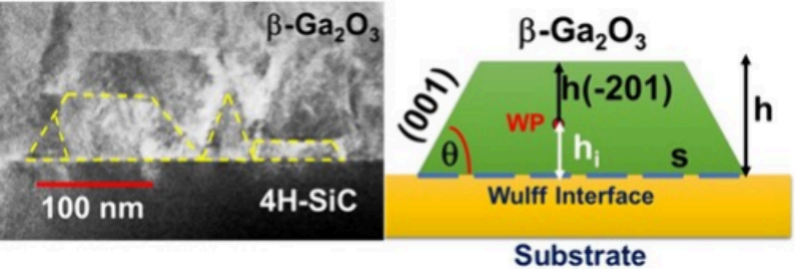

Heteroepitaxy of gallium oxide (Ga_2_O_3_) is gaining popularity to address the absence of p-type doping, limited thermal conductivity of Ga_2_O_3_ epilayers, and toward realizing high-quality p-n heterojunction. During the growth of β-Ga_2_O_3_ on 4H-SiC (0001) substrates using metal–organic chemical vapor deposition, we observed formation of incomplete, misoriented particles when the layer was grown at a temperature between 650 °C and 750 °C. We propose a thermodynamic model for Ga_2_O_3_ heteroepitaxy on foreign substrates which shows that the energy cost of growing β-Ga_2_O_3_ on 4H-SiC is slightly lower as compared to sapphire substrates, suggesting similar high-temperature growth as sapphire, typically in the range of 850 °C–950 °C, that can be used for the growth of β-Ga_2_O_3_ on SiC. A two-step modified growth method was developed where the nucleation layer was grown at 750 °C followed by a buffer layer grown at various temperatures from 920 °C to 950 °C. 2θ–ω scan of X-ray diffraction (XRD) and transmission electron microscope images confirm the β-polymorph of Ga_2_O_3_ with dominant peaks in the (−201) direction. The buffer layer grown at 950 °C using a “ramp-growth” technique exhibits root-mean-square surface roughness of 3 nm and full width of half maxima of XRD rocking curve as low as 0.79°, comparable to the most mature β-Ga_2_O_3_ heteroepitaxy on sapphire, as predicted by the thermodynamic model. Finally, the interface energy of an average Ga_2_O_3_ island grown on 4H-SiC is calculated to be 0.2 J/m^2^ from the cross-section scanning transmission electron microscope image, following the Wulff-Kaishew theorem of the equilibrium island shape.

## Introduction

The ever-advancing landscape of high-power commercial applications and the pursuit of a net-zero society have driven significant evolution in semiconductor materials. Gallium oxide has emerged as a potential game-changer, boasting an ultrawide bandgap (4.5 to 4.9 eV) and an impressive critical electric field of 8 MV/cm, making it a standout candidate.^1−3^ Ga_2_O_3_ offers distinct advantages over established materials like Gallium Nitride (GaN) and Silicon Carbide (SiC) due to its significantly higher critical electric field. As GaN and SiC technologies mature, Ga_2_O_3_ stands out as a favored contender to provide more efficient solutions for high-voltage commercial power applications. While five stable Ga_2_O_3_ polymorphs are known (α, β, γ, δ, and κ), recent years have witnessed a surge in interest toward the most stable β polymorph due to its unique and potentially game-changing properties.^28^ β-Ga_2_O_3_ is presently the most considered for power electronic applications as it is the most thermodynamically stable phase; however, the metastable α and κ polymorphs should not be ignored; these exist under specific conditions and can offer advantages. For example, α-Ga_2_O_3_ being a corundum phase, boasts a hexagonal crystal structure and the widest bandgap (5.2 eV) among all Ga_2_O_3_ polymorphs. This exceptional bandgap makes it highly desirable for power devices operating at high voltages and temperatures.^41,42^ On the other hand, κ-Ga_2_O_3_ possesses an orthorhombic crystal structure, can offer piezoelectric properties similar to how a GaN electronic device work, and can be deposited on commercially available (0001)-oriented sapphire substrates using MOVPE.^43^ Nonetheless, metastable α, κ(ε), and γ-Ga_2_O_3_ films seek a lower-energy state by transforming into the more thermodynamically favorable β-Ga_2_O_3_ structure by rearranging their atoms to minimize the overall energy. More details on this can be found elsewhere.^45,46^

However, challenges persist, including the absence of p-type doping and limited thermal conductivity, leading to excessive heating in high-power devices that can degrade performance, reliability, and lifetime.^2,3^ The integration of Ga_2_O_3_ with high thermal conductivity substrates, such as silicon carbide (SiC) and diamond, has gained prominence for advanced electronic and power device applications to tackle these limitations. Two primary methods, epitaxial growth and direct bonding, have emerged as crucial techniques for this integration. Notable achievements include the formation of rectifying p–n junctions through low-temperature direct bonding, as demonstrated by Sittimart et al.,^4^ and the development of β-Ga_2_O_3_ field-effect transistors (FETs) on diamond substrates using Ga_2_O_3_ nanomembranes, as illustrated by Noh et al.^5^ Moreover, Song et al. reported the creation of Ga_2_O_3_/4H-SiC composite wafers through a fusion bonding method, leading to metal-oxide-semiconductor field-effect transistors (MOSFETs) with significantly reduced channel temperatures and an impressive power figure of merit.^6^ However, challenges exist in direct bonding, as Cheng et al. pointed out the limitation of weak van der Waals bonding between the substrate and the Ga_2_O_3_ layer; this hinders the full utilization of the thermal conductivity in diamond and SiC substrates.^7^ Recent successes in this domain include the high-quality epitaxial growth of Ga_2_O_3_ on diamond achieved by Nandi et al.^8^ and Karim et al.^9^ Besides, Girolami et al. recently demonstrated growth of κ-Ga_2_O_3_ on polycrystalline diamond substrates.^44^ Furthermore, several reports have outlined the successful heteroepitaxy of Ga_2_O_3_ on SiC substrates, opening new avenues for enhanced device performance and thermal management.

Recent research, such as Hrubisak et al.’s work on liquid injection metal–organic chemical vapor deposition (MOCVD) growth of monoclinic β-Ga_2_O_3_ films on 4H-SiC,^10^ Hu et al.’s achievement of step-flow growth of β-Ga_2_O_3_ films on off-axis 4H-SiC via LPCVD,^11^ and Xia et al.’s extension to hexagonal phase-pure ε-Ga_2_O_3_ films on 6H-SiC using MOCVD,^12^ has contributed to the understanding of Ga_2_O_3_ growth dynamics on high thermal conductivity substrates. However, improvements are still needed to meet commercial standards, exemplified by commercial GaN on Si heteroepitaxy, where epilayer quality routinely exhibits XRD rocking curve fwhm values in the range of 0.1°–0.11°.^13,14^ The best quality of β-Ga_2_O_3_ on foreign substrates has been obtained on c-plane sapphire substrates, typically with fwhm values ranging from 0.6°–1°.^15−24^ Further improvement in layer quality can be achieved by using offcut substrates. A better understanding of growth dynamics is crucial to achieve the highest quality of Ga_2_O_3_ on high thermal conductivity substrates, such as diamond and SiC, for commercial applications.

MOCVD growth is controlled by both thermodynamics at equilibrium and growth kinetics at steady-state. While there are established thermodynamic models for Ga_2_O_3_ homoepitaxy, the field of Ga_2_O_3_ heteroepitaxy is still in its nascent stages, and as of now, there is no well-established thermodynamic model for this process. When growing Ga_2_O_3_ on a substrate with a different lattice constant, significant strain arises in the Ga_2_O_3_ film. The strain energy increases as the film thickness grows.^31−33^ Once the mismatch surpasses a specific threshold, usually around 1–2%, it leads to the formation of 3D islands of Ga_2_O_3_ through elastic deformation, easing some of the strain caused by the lattice mismatch and reducing the stored elastic energy. Another option involves the creation of misfit dislocations to accommodate the strain.^25^ It is essential to note that the formation of these Ga_2_O_3_ nucleation sites comes at the expense of increased surface energy and interface energy.^29^ In a two-component system like this, where the materials are immiscible, multiple energy factors must be taken into account. These factors encompass the energy related to the free surface of the substrate, the energy of the overlying material’s surface, the interfacial energy at the Ga_2_O_3_-substrate boundary, and the strain energy. Moreover, during the growth process, the chemical potential of adatoms on the substrate also plays a crucial role. The interaction between the chemical potential of adatoms and the substrate-mediated strain adds further layers of complexity to this intricate process.

### Thermodynamic Model

**Figure 1:**
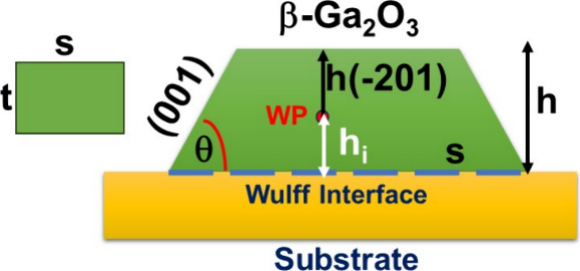
Formation of a 3D Ga_2_O_3_ island on a foreign substrate according to the Wulff-Kaishew (WK) construction. The blue dash line indicates the Wulff interface, and the red dot indicates the Wulff point.

Let us consider the initial nucleation phase of β-Ga_2_O_3_ on a foreign substrate, assuming a Volmer–Weber growth mode where the substrate surface energy is lower than the epilayer surface energy.^34^ This phase begins with the formation of a trapezoidal 3D island with a length denoted as *s* and a width as *t* as illustrated in Figure 1. As the thickness *h* of the nucleation layer increases, the island sidewall will incline at an angle θ to the substrate surface. Both the substrate and the Ga_2_O_3_ island possess different surface energies, represented as Γ_s_, Γ_t_, and Γ_e_, i.e., the substrate surface energy, the surface energy of the top epilayer, and the surface energy of the inclined sidewall, respectively. In terms of surface energy minimization, the likelihood of the crystallographic orientation of Ga_2_O_3_ side-wall is (001) B with a surface energy of 2.37 J/m^2^ (Γ_e_) as compared to the (−201) top surface with a surface energy of 2.67 J/m^2^ (Γ_t_). This corresponds to a value of 50° for θ, a balance between surface and interface energy. In general, for isotropic elasticity, the energy of an island per unit volume can be written as^27^
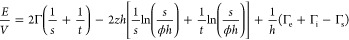
1where Γ is the relative surface energy and can be expressed by 2Γ = 2Γ_e_ csc θ – (Γ_t_ + Γ_i_ – Γ_s_)cot θ, and Γ_i_ is the nonzero interface energy,  with *G* the shear modulus and *v* the Poisson’s ratio. The in-plane stress *σ*_b_ can be determined using^26^ where *σ*_*xx*_ and *σ*_*yy*_ denote the *xx* and *yy* stress components, respectively, *E* represents the Young’s modulus, and *m* signifies the lattice mismatch.

The formation of a 2D layer or 3D islands relies on the equilibrium among the surface energy of the substrate and the top epilayer, along with the interface energy, and depends on the wetting factor *W* where
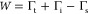
2In the context of 2D growth, i.e., Stranski-Krastanov growth, *W* is negative; conversely, positive *W* values signify a 3D island, or Volmer–Weber growth. However, adhesion fails entirely when the *W* ≥ 2Γ_t_.

For a thorough comparison of heteroepitaxial growth of Ga_2_O_3_ on different substrates, assessing the interface energy is crucial, although not straightforward. The Wulff-Kaishew (WK) theorem aids in estimating the interface energy, considering the equilibrium island shape, as shown in Figure 1.^39^ In equilibrium, the positioning of the stable 3D island’s shape balances the interface energy such that
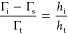
3with *h*_i_ representing the distance of the Wulff point from the interface, and *h*_t_ signifies the distance of the island surface from the interface (also known as Wulff interface), as shown in Figure 1. The Wulff point is defined by a point location in the crystal where the distance of the crystal facet from that point is proportional to the surface energy of that facet, as shown in Figure 1. We can now consider a set of boundary conditions in order to evaluate the energy cost of a Ga_2_O_3_ island in equilibrium.

Let us discuss first the onset of 2D growth where *h*_i_ = −*h*_t_; i.e., Γ_i_ – Γ_s_ = −Γ_t_. In this case, the wetting factor *W* is zero and the interface energy is now negative since −Γ_s_ < Γ_t_. Any value of *h*_i_ closer to the interface results in a positive wetting factor, leading to 3D island growth. The second boundary condition arises when *h*_i_ = 0; i.e., the Wulff point lies on the interface itself. In this scenario, the interface energy equals to the substrate surface energy. Furthermore, *W* is equal to 2Γ_t_ when *h*_i_ = *h*_t_. For any values of *h*_i_ > *h*_t_, adhesion fails entirely.

Hence, for the Volmer–Weber growth mode, the inequality Γ_s_ – Γ_t_ < Γ_t_ < Γ_t_ + Γ_s_ holds. Notably, in the Volmer–Weber growth, where the substrate surface energy is less than the epilayer surface energy, a negative interface energy implies a higher base-to-height ratio of the grown island, fostering lateral growth. In practical scenarios, for any 3D island growth, Γ_i_ > 0. Therefore, for Volmer–Weber growth, we can assume:


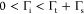
4A lower interface energy promotes greater lateral growth compared to vertical growth, enhancing a smoother surface morphology. Conversely, the interface energy closer to the maximum limit, i.e., Γ_t_ + Γ_s_, mostly results in smaller islands in size with a rougher surface.

Applying these boundary conditions to eq 1, we now can calculate the energy cost of a Ga_2_O_3_ island of unit volume on a foreign substrate. Considering two boundary conditions: first, *h*_i_ = 0, i.e., Γ_i_ = Γ_s_ as discussed above, the calculated energy cost (E/V) amounts to 6.26 kJ/cm^3^ on sapphire and 6.14 kJ/cm^3^ on 4H-SiC. Thus, maintaining growth equilibrium on sapphire (001) and 4H-SiC (001) requires an interface energy of 1.85 and 1.7 J/m^2^, respectively. Second, when *h*_i_ = *h*_t_, implying Γ_i_ = Γ_t_ + Γ_s_, the energy cost amounts to 12.1 kJ/cm^3^ on sapphire and 11.9 kJ/cm^3^ on 4H-SiC, leading to interface energies of 4.52 and 4.37 J/m^2^ on sapphire and 4H-SiC substrate, respectively. Table 1 provides a summary of all of the key parameters utilized in these calculations.

**Table 1:** Parameters Used to Calculate the Total Energy Cost of a Ga_2_O_3_ Island

Parameters	β-Ga_2_O_3_	c-plane sapphire	4H-SiC	Diamond
Surface energy (J/m^2^)	2.67 (−201), 2.37 (001B)	1.85	1.7	6.6 (100)
				4 (110)
				2.7 (111)
Shear modulus (GPa)	80	145	367	508.3
Poisson’s ratio	0.31	0.29	0.2	0.2
Lattice mismatch (%)	0	6.25	3.25	3.06

These calculations suggest that a higher energy cost results in higher interface energy and thus smaller island sizes. Interestingly, Ga_2_O_3_ islands on 4H-SiC require slightly less interface energy than those on sapphire. Extending this analysis to diamond substrates such as (111), (110), and (100), the surface energy increases here from 2.7 and 4 to 6.6 J/m^2^ corresponding to the (111), (110), and (100) surfaces, respectively.^30,40^ Therefore, it is possible that strained Ga_2_O_3_ epilayers completely wet the diamond substrate, following Stranski-Krastanov growth, at least initially since the surface energy of diamond is significantly higher than the β-Ga_2_O_3_ (−201) surface energy. Further analysis provides energy costs of −973, −99.8, and 303 J/cm^3^ on (100), (110), and (111) diamond substrates, respectively. Notably, despite the lowest surface energy of the diamond (111) substrate, the positive energy cost suggests that our assumption may not favor the (001) side wall facet inclined to the (−201) plane, particularly on the diamond (111) surface.

From the thermodynamic analysis conducted thus far, it is evident that the growth of Ga_2_O_3_ islands on sapphire and 4H-SiC should follow a similar pattern, characterized by Volmer–Weber growth with very similar energy costs and interface energies. Therefore, it is thermodynamically favorable to employ high-temperature Ga_2_O_3_ growth on 4H-SiC, akin to the methods widely utilized on sapphire substrates. However, the growth of Ga_2_O_3_ on low-angle diamond substrates exhibits a preference for Stranski-Krastanov growth, necessitating a distinctly different growth approach compared to Ga_2_O_3_ growth on sapphire and 4H–Si as reported elsewhere.^8^ The subsequent section elaborates on the epitaxial growth and characterization of β-Ga_2_O_3_ on 4H-SiC, which is in line with the predictions derived from the preceding thermodynamic analysis.

### MOCVD Growth of β-Ga_2_O_3_ on 4H-SiC

All the growths in this study were conducted on commercially available semi-insulating 4H-SiC substrates (0001) using an Agnitron Agilis 100 metal–organic chemical vapor deposition (MOCVD) system. Typically, β-Ga_2_O_3_ on SiC that are reported so far are grown within a temperature range of 650 °C–750 °C.^10−12^ Nevertheless, our thermodynamic modeling indicated that the energy cost for growing phase-pure β-Ga_2_O_3_ on 4H-SiC is quite similar to that on sapphire. Therefore, the likelihood of nucleation is expected to be comparable. Notably, high-quality β-Ga_2_O_3_ growth on sapphire is often reported at temperatures exceeding 800 °C.^15,16^ However, one of the challenges in growing β-Ga_2_O_3_ on SiC substrate with a pure oxygen source at high temperature is the formation of an amorphous SiO_2_ layer on the growth surface that can potentially result in a poor crystal quality. Si-terminated 4H-SiC/β-Ga_2_O_3_ interface is highly sensitive to oxygen due to its lowest migration energy. O-terminated β-Ga_2_O_3_ in the (−201) direction and Si-terminated 4H-SiC in the (0001) direction offer the lowest relaxation energy and thus the highest stability by forming covalent bonds between Si–O at the interface, depending on how the oxygen atoms migrate on the 4H-SiC surface during the initial growth phase of β-Ga_2_O_3_. Therefore, the Si-terminated SiC surface is highly reactive to the oxygen.

**Figure 2:**
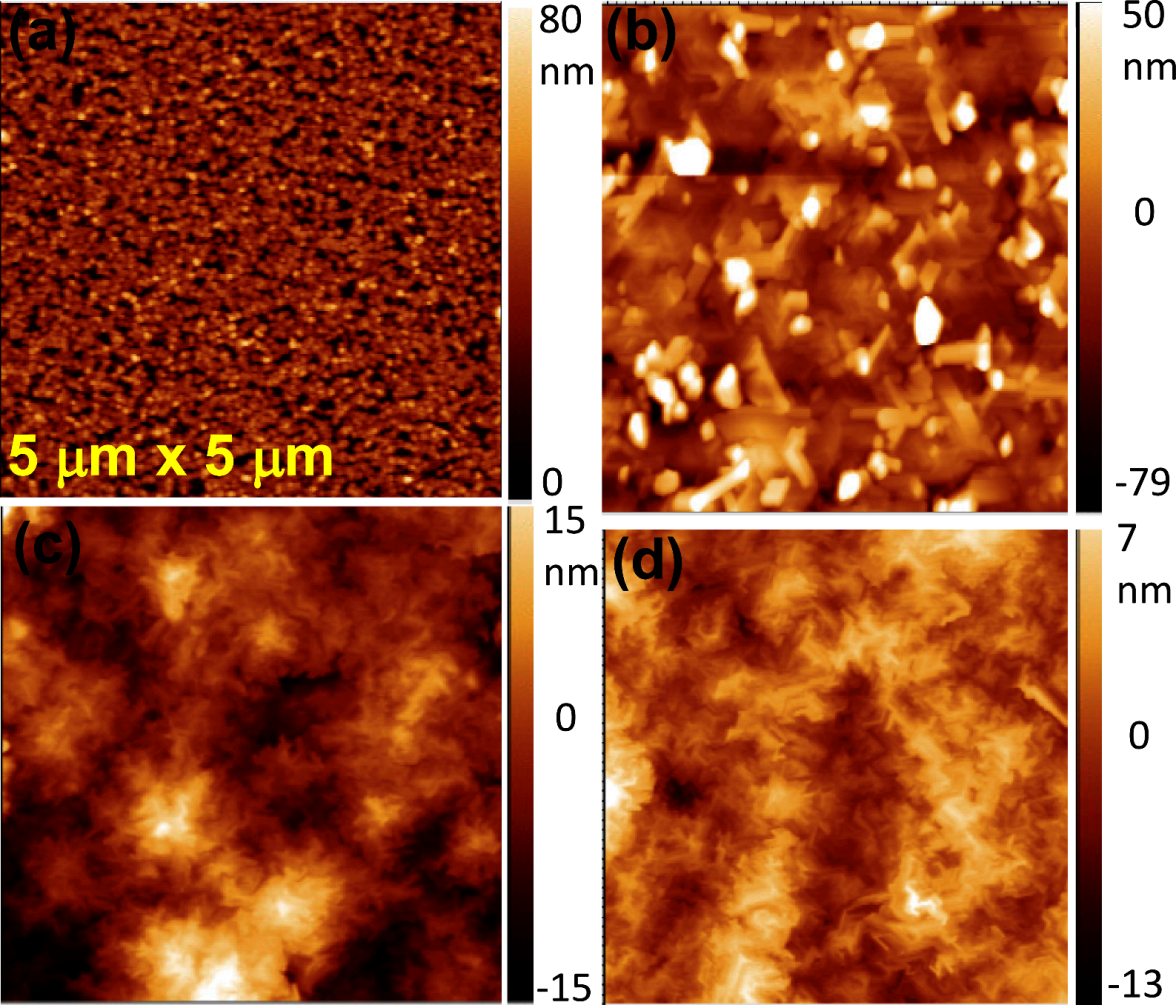
AFM surface morphology of Ga_2_O_3_ layers grown under different conditions. (a) Surface morphology of Ga_2_O_3_ layer grown at 750 °C throughout; (b) surface morphology of S1, displaying full coalescence and the presence of misoriented grains; (c) S2 presenting a fully coalesced surface devoid of nanoparticles; and (d) S3, exhibiting a smooth surface with RMS roughness of 3 nm, signifying enhanced quality.

Therefore, we adopted a two-step modified growth method where the nucleation layer was grown at 750 °C to prevent the surface oxidation of the SiC substrate, followed by approximately 350 nm of buffer layer grown at various temperature from 750 °C to 950 °C. Four samples, namely, S0, S1, S2, and S3, were compared to understand the effect of growth parameters on the layer quality. S1 and S2 are identical except for the bulk layer where the growth temperature was 920 °C and 950 °C, respectively. As shown in Figure 2(a), the sample S0 grown at 750 °C throughout exhibits a surface morphology as expected from the thermodynamic model, closely resembling those reported by others at similar growth temperature. S1 shows a partly coalesced surface with a few misoriented grains or nanocrystallites as shown in Figure 2(b). Facet edges approximately 60° and 120° in different directions are also visible, which signify different pseudohexagonal domains. In contrast, S2 forms a fully coalesced surface without any visible nanocrystallites on the surface as can be seen in Figure 2(c), suggesting a high quality of the grown layer. Furthermore, AFM scans shown in Figure 2(b,c) reveal an average root-mean-square (RMS) roughness of 16 nm and 7 nm in S1 and S2, respectively, across a 5 μm × 5 μm area. Rather than employing a conventional method of growing a thin nucleation layer at a constant lower temperature, we introduced a novel approach called the ’ramp-growth’ technique in S3. In this technique, the nucleation layer’s growth initiates at 780 °C, and the temperature gradually was increased to 950 °C over the course of 3 min, i.e., a ramp rate of 56 °C/min while the growth process is ongoing to mimic the high-temperature β-Ga_2_O_3_ growth on sapphire by avoiding SiO_2_ formation. Subsequently, the buffer layer is grown at 950 °C, similar to S2. As can be seen in Figure 2(d), the fully coalesced surface of S3 shows a roughness down to 3 nm, lowest among all the samples. It is noteworthy that the temperature ramp rate slightly affects the surface morphology of the following epilayer and is also influenced by various growth conditions. The temperature ramp rate of 56 °C/min was optimized at a rector pressure of 40 Torr, a TEGa flow of 26 μmol/min, and O_2_ flow of 800 sccm in terms of full width at half-maximum (fwhm) obtained from the ω-scan along the (−402) plane and the AFM surface morphology. A post growth cooling rate of 70 °C/min was used for all the samples studied here. As outlined by Girolami et al., the post growth cooling rate may influence the formation of macroscopic defects and the overall layer quality; it was not studied systematically for the samples under study.^35,44^

## Results and Discussion

**Figure 3:**
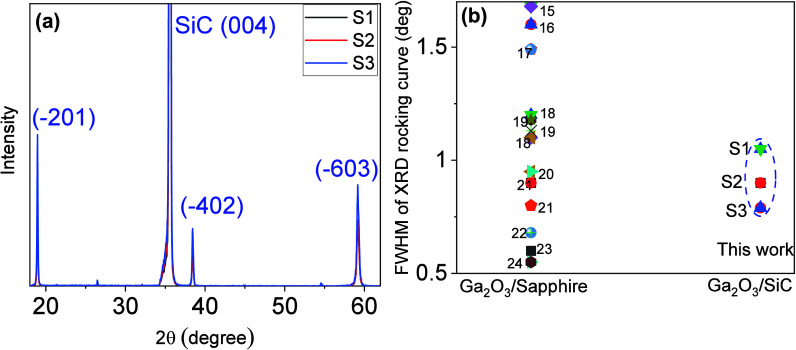
(a) XRD 2θ–ω scan in linear scale of samples S1, S2, and S3, revealing distinct peaks at 18.9°, 38.4°, and 59.2°, corresponding to the (−201), (−402), and (−603) crystallographic planes, respectively, of the beta polymorph of Ga_2_O_3_; (b) benchmarking reported fwhm of Ga_2_O_3_ grown on sapphire, from refs as indicated in the figure and SiC including samples S1, S2, and S3 in this study.

Figure 3 shows the XRD 2θ–ω scans of the grown samples. All the samples display consistent peaks at 18.9°, 38.4°, and 59.2°, which can be attributed to the (−201), (−402), and (−603) crystallographic planes, respectively, of the beta polymorph of Ga_2_O_3_. It is evident that the (−201) plane is the predominant growth direction on the SiC (001) plane, consistent with previously reported heteroepitaxial Ga_2_O_3_ on sapphire, 4H-SiC and diamond. Furthermore, the full width at half-maximum (fwhm) obtained from the ω-scan along the (−402) plane, which serves as an indicator of material quality, is presented in Figure 3(b). S1 and S2 exhibit fwhm values of 1.02 degrees and 0.9 degrees, respectively. In contrast, S3 boasts the lowest fwhm at 0.79°, the lowest among previously reported β-Ga_2_O_3_ samples on 4H-SiC and placing it on par with some of the highest-quality Ga_2_O_3_ samples grown on sapphire.

**Figure 4:**
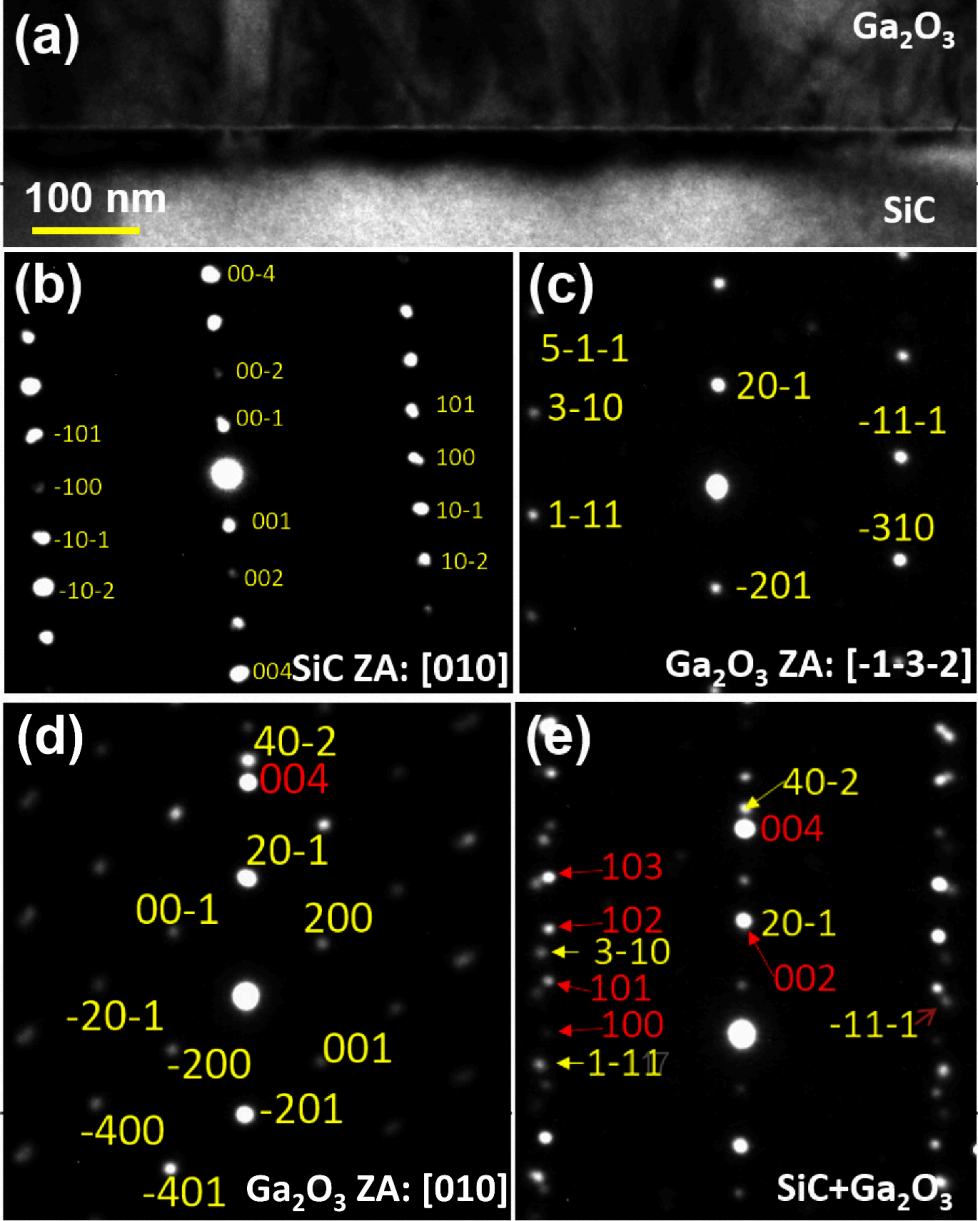
(a) Edge-on bright-field TEM image of S3. Selected area electron diffraction (SAED) reflections of S3 with (b) SiC aligned to the [010] zone axis, (c) β-Ga_2_O_3_ aligned with the [−1–3–2] zone axis, (d) β-Ga_2_O_3_ aligned with the [010] zone axis, and (e) reflection from β-Ga_2_O_3_ and SiC, aligned with [−1–3–2], with the β-Ga_2_O_3_ interfacial plane parallel to [010] of SiC.

In order to find the epitaxial relationship of the grown layer with 4H-SiC, we further looked into the selected area electron diffraction (SAED) patterns from β-Ga_2_O_3_-on-SiC. Figure 4(a) shows the edge-on bright field TEM image of S3. Panels (b), (c), (d), and (e) show the corresponding SAED patterns originating from SiC aligned with [010] zone axis, Ga_2_O_3_ aligned with [−1–3–2] zone axis, Ga_2_O_3_ aligned with [010] zone axis, and Ga_2_O_3_ and SiC together aligned with [−1–3–2] of Ga_2_O_3_ interfacial plane parallel to [010] of SiC, respectively. For the SiC reflections, depicted in Figure 4(b), the reflections should be multiples of (001) along the *c*-axis in the electron diffraction pattern with (004) dominant, as this is the allowed reflection. Reflection from (001), (002), and (003) appears through double diffraction. In the perpendicular direction, diffractions from (100), (101), (10–2), etc., are allowed and visible in Figure 4(b). Note that the strong reflection in Figure 4(d) along the (−201) row is the SiC (004) reflection; (002) should be weak or effectively absent along with (001) and (003), which need double diffraction. Taking the relative spacing into account, the (40–2) reflection from Ga_2_O_3_ and the (004) reflection from SiC, both spots in Figure 4(e), should be adjacent to the (40–2) reflection and will be strongly excited when there is any SiC in the selected area, which is presumably the bright spot due to the (004) reflection in Figure 4(d) as well. The reflection from SiC (002) in Figure 4(e) just below Ga_2_O_3_ (20–1) is rather weak as can be seen also in Figure 4(a). Therefore, the relative diffraction of SiC and Ga_2_O_3_ establishes an epitaxial relationship of [010]/[−1–3–2] (−201) β-Ga_2_O_3_ || [010] (001) 4H-SiC.

**Figure 5:**
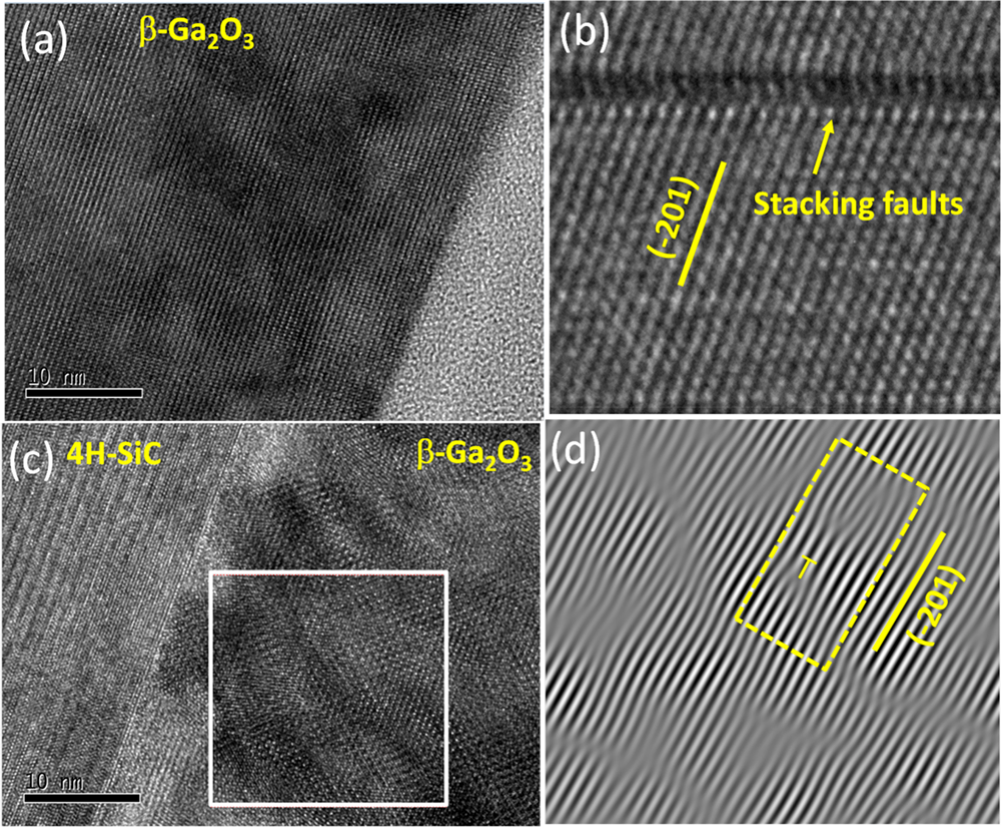
(a) HRTEM image of Ga_2_O_3_ layer in S3, (b) stacking faults in the grown layer, (c) illustrating the Ga_2_O_3_–SiC interface, and (d) an inverse FFT image of the square region in (c) showing edge-type dislocations.

Figure 5(a) shows an HR-TEM image of the grown Ga_2_O_3_-on-SiC epilayer of sample S3. The strain relaxation of Ga_2_O_3_ nucleation on SiC primarily hinges on minimizing energy, as captured by the thermodynamic model discussed above by considering factors like strain–stress (*σ*_b_) and surface energy (Γ). Despite a sharp interface, as can be seen in Figure 5(c), localized defects near the SiC/Ga_2_O_3_ interface and the formation of edge-type dislocation can be seen during the nucleation phase of the epilayer due to strain relaxation. Stacking faults (SF) form, associated with partial dislocations as visible in Figure 5(b,d). The presence of compressive or tensile strain significantly impacts the dislocations that emerge, leading to stacking faults. Interestingly, regardless of whether strain is compressive or tensile, both systems should behave similarly in energy minimization. This is because strain contributes to the thermodynamic model with a squared term (i.e., σ_b_^2^ in eq 3), rendering the sign of strain (hence compressive or tensile) irrelevant.

**Figure 6:**
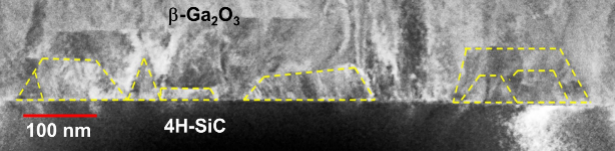
STEM cross-section image of the Ga_2_O_3_ epilayer on the 4H-SiC substrate (S3). The yellow dashed lines indicate the shapes of the 3D islands during the initial deposition of Ga_2_O_3_.

As discussed earlier to estimate the energy cost of island growth, it is important to correctly estimate the interface energy. The proposed thermodynamic model gives us a range of interface energy which varies in the range 0 < Γ_i_ < Γ_t_ + Γ_s_ (eq 4). Fortunately, experimental estimation of the interface energy by using the same WK theorem is possible.^36−38^ In Figure 6, a STEM cross-section image of the sample S3 suggests an initial growth phase with 3D-like islands, followed by lateral coalescence during continuous layer growth. As depicted in Figure 6, these Ga_2_O_3_ islands can be approximately fitted with trapezoidal island shapes, as predicted by the WK theorem in Figure 1. The height-to-base ratio of the 3D nucleation islands varies among different individual islands significantly, ranging from 0.23 to 0.44, with a mean value of 0.32. This ratio represents how tall the island is relative to its base dimension. Additionally, the angles formed between the side walls and the top surface of these islands exhibit some variance due to partial coalescence during continuous growth. Most commonly, these angles fall within the range of 123°–135°, with a mean value of 128°. Although the coalescence of islands may affect the precise geometry, these results are consistent with our assumption of (001) side walls with an angle of approximately 130° to the top surface as considered in the thermodynamic model.

As the side wall could be either (001)A or (001)B, it remains indeterminate from Figure 6 which observed (001) facet has A or B orientation; let us assume (001 B) due to its lower surface energy relative to the top (−201) surface.

Using simple geometry and WK theorem, one can write
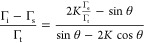
5where *K* = *h*/*b*, *h* and *b* are the height and the base of the trapezoid, respectively.^39^ Taking the average value of *K* as 0.32 and considering a (001)B side wall with an angle of 130° with the top (−201) plane, which translates to θ = 50° (the angle between the side-wall facet), we calculate the ratio of = −0.56. Consequently, the interface energy of an average Ga_2_O_3_ island on 4H-SiC is calculated to be 0.2 J/m^2^. Remarkably, this value closely aligns with the lower limit of the interface energy predicted by eq 4, thereby validating our thermodynamic model.

## Conclusion

In summary, the success of Ga_2_O_3_ growth on foreign substrates is influenced not only by lattice mismatch but also by the differences in surface energy. A thermodynamic analysis indicates that the growth of Ga_2_O_3_ islands on both sapphire and 4H-SiC follows a comparable pattern, characterized by Volmer–Weber growth with nearly identical energy costs and interface energies. Hence, employing high-temperature Ga_2_O_3_ growth on 4H-SiC, similar to widely adopted methods on sapphire substrates, is thermodynamically advantageous. This is successfully illustrated here. To prevent SiC substrate oxidation at high temperatures, however, in contrast to growth on sapphire, a two-step modified growth method was employed. It involved a nucleation layer grown at 750 °C, followed by a buffer layer at various temperatures from 750 °C to 950 °C. Varying the buffer layer temperature resulted in partly coalesced surfaces with misoriented grains at 920 °C and fully coalesced surfaces at 950 °C. XRD analysis and HRTEM images confirmed the phase-pure β-polymorph of Ga_2_O_3_. Furthermore, a new ’ramp-growth’ technique was introduced, starting the nucleation layer at 780 °C and gradually increasing the temperature to 950 °C within 3 min. This approach significantly improved surface quality, reducing roughness to 3 nm and lowering the fwhm to 0.79°. These results are comparable to most matured β-Ga_2_O_3_ heteroepitaxy on sapphire, promising potential for high-voltage device fabrication with improved thermal properties and realizing a high-quality p-n heterojunction.
